# Taveau’s Pâte D’Argent Not the First Amalgam

**Published:** 1898-12

**Authors:** William H. Trueman

**Affiliations:** Philadelphia


### TAVEAU'S PÂTE D'ARGENT NOT THE FIRST AMALGAM.

BY WILLIAM H. TRUEMAN, D.D.S., PHILADELPHIA.

THERE seems to be an impression among dental writers in this country that the use of amalgam for filling cavities of decay in human teeth was first suggested by O. Taveau, a dental writer of Paris, about 1826. To show that this is an error, I quote those paragraphs in his work upon which alone the claim may be founded. They are found in a work entitled "Nouvelle Hygiène de la Bouche," published at Paris in 1843; the fifth edition, rewritten and enlarged, of a work first published by him in 1826. On page 232 he enters upon the discussion of materials for filling teeth, giving the advantages and disadvantages of those then in use, and on page 236 introduces the "pâte d'argent," or silver paste, as follows:

"To obviate all these inconveniences, I have used the last seven or eight years, with incontestable advantage, a paste which I have named pâte d'argent, the composition of which I at once communicated to our *confrères.* It is the same as that which an English dentist has since pretended to have imported to Paris, giving out that it was something new, and which he improperly proposes to designate by the name 'mineral succedaneum,' a designation which, to say the least, is nonsense, for it does not indicate either its nature or its use. This paste is made of pure silver and mercury.

"To prepare it, we saturate the silver, reduced to a very fine powder, with a given quantity of mercury, rubbing them together until they are perfectly incorporated. We then use it as it is, or, to make it harder, we press strongly the mass enclosed in the skin of a kid deprived of its epiderm, in order to extract all the mercury. The material we obtain by this process is a species of hard paste, but soft enough to yield easily to pressure of the fingers.

"We employ this preparation cold, making it, with a plugger, penetrate and fill the cavity of decay and all its irregularities as perfectly as do the materials used in leaves. The mercury it contains is evaporated by the heat of the mouth alone, and this during the course of the following day, the silver remaining united in one piece within the cavity of the tooth, filling all its anfractuosities, and becoming compact and solid in every part of the cavity. This new means of filling is incontestably superior to all others which we employ at the present time, because, first, the amalgam makes a better filling without requiring so much packing as do the metals in leaves; also, by its use we avoid the application of heat, necessary with the Darcet metal; and, finally, it becomes so hard after a little time, as proved by our experience, that when in the tooth it has all the requisites of a perfect filling.

"The silver paste does not shrink, as we might fear, from the evaporation of the mercury which remains after the surplus has been expressed through the kid-skin, which process leaves a portion almost inappreciable. As for the fears we may have of the action of this portion of mercury upon the teeth, they are absolutely illusive; this we say in reply to M. Lefoulon, seeing that the quantity of mercury which remains is very much less than is contained in the Darcet metal as modified by Regnard, to which it is infinitely superior in hardness."

From the data here given we may reasonably infer that Taveau first used the silver paste about 1835 at the earliest, at which time we have abundant evidence that under various names it was well known and widely used. This work, published in 1843, was, as we have before said, the fifth edition of a work originally published by the same author in 1826; and the question naturally arises, Are these references to silver paste found in any of the earlier editions?

The first edition, entitled "Hygiène de la Bouche," or a "Treatise on the Care necessary to keep the Mouth in Order and to conserve the Teeth," dated at Paris, 1826, is a 12mo volume of 208 pages, written for the public, and dedicated to mothers of families. The only reference to materials for filling cavities of decay is found on page 159, where the writer says, "The manner of plugging the teeth (plomber les dents) is entirely different to-day from what it was some years ago, when we employed for this purpose lead or gold in leaves, which were introduced by means of a stylet. We now use a hard metal, which, on the approach of a heated iron, not very hot, and without causing the least pain, promptly fuses and spreads within the cavity, filling it in every part. This new method has the immense advantage over the old of, first, filling more exactly the cavity of decay; second, presenting an exterior surface hard and polished; third, of not giving any odor to the mouth; fourth, and finally, it can be quickly done without causing the least pain."

We here recognize the fusible metal of Newton, or the French Darcet metal, composed of three parts of tin, five of lead, and eight of bismuth.

The second edition, also dated 1826, I have not seen.

In the third edition, corrected and enlarged to 276 pages, dated Paris, 1828, beginning on page 198, we find precisely the same words used; a few lines are added, stating that, while the heat necessary for fusing the metal may be noticed for a moment, it is not sufficient to cause the least danger, and that many persons do not notice it at all.

In the fourth edition, dated 1833, enlarged to 288 pages, we find on page 198 precisely the same words as are found in the third edition. In none of these is the silver paste mentioned

We have further evidence upon this point in a little pamphlet written by Taveau, dated Paris, 1837 (the first edition), entitled

"Notice of a Cement for arresting Caries of the Teeth, and offering a New Mode of Treatment for conserving those which are attacked, without having Recourse to the Extraction of these Precious Organs." [1]

He here speaks of the use of gold, tin, and lead, in very thin leaves, and of the fusible or Darcet metal, used for filling cavities of decay. Not a word do we find of "pâte d'argent," which surely would have been mentioned if he was at that time in the habit of using it. He was not a man to keep such things to himself, but promptly, as he tells us he felt it his duty to do, he published, that others might enjoy them with him, any new discoveries that he made. In this pamphlet he refers to the fact that the fillings then in use merely replaced lost tissue; they simply protected the diseased part of the tooth from irritating and destructive agents; they were merely mechanical expedients, and were not in themselves curative. He refers to the use of the actual cautery, of acids, of the caustic alkalies, and of nitrate of silver for arresting the progress of caries, and proposes in place of these, as a preparatory filling, a cement composed of anhydrous alum, alcohol, ether, and gum mastic.[2] This composition, he says, "like hydraulic cement with water, hardens prodigiously in a few hours." He asserts that "two or three applications—more frequently only one—is sufficient to completely destroy the caries and conserve the tooth without pain." There was much confusion in the use of terms among dentists the world over when this was written. Reading between the lines, I am impressed that his proposed cement was intended to produce a species of pulp-mummification, and was most likely employed in cases of either near approach to or actual pulp exposure, not to arrest the progress of caries, as we now understand this expression, but to obtund the sensitiveness of the cavity, so that a filling could be inserted. I have referred to this pamphlet more at length, because its title would naturally lead one to suppose that it treated of cements for filling teeth. It does not, however. It was written for the sole purpose of calling attention to the use of the alum and mastic compound for use in sensitive cavities, preparatory to filling them with other material.

---

[1] Notice sur un ciment oblitérique pour arrêter et guérir la caries des dents, offrant un mode nouveau de traitement pour conserver celles qui en sont atteintes sans avoir recours à l'extraction de ces précieux organes. By Ore Taveau. Paris, 1837.

[2] Sursulfate d'alumine anhydre et l'extrait alcoholique et éthéré du pistacia lentiscus de l'ile de Chio.

The evidence presented warrants, I think, the presumption that Taveau's own statement in the fifth edition of his "Hygiene of the Mouth," published at Paris in 1843, fixes the date of his first use of the silver paste years after it had been used by others, and that crediting him with having first introduced amalgam to the dental profession is an error. We will now ask, How did this error originate?

In 1841, J. Lefoulon, a dentist of Paris, published a treatise upon the "Theory and Practice of Dental Surgery." This was translated into English by Dr. Thomas E. Bond, Jr., and published at Baltimore in 1844 by the American Society of Dental Surgeons. In this treatise Lefoulon refers to the silver paste as the invention of Taveau,[1] and we can readily imagine that the American dental public, who had been so recently excited by the advent of the Crawcours and their royal mineral succedaneum, an amalgam supposed to be a French invention, would at once accept this version of its origin. Shortly before the publication of Bond's translation of Lefoulon, the fifth edition of Taveau's "Hygiène de la Bouche" appeared, containing the account of the "pâte d'argent" I have quoted; and we may readily imagine some dentist seeing this, and, being curious to know when this work first appeared, would naturally consult a dental bibliography, possibly that found in Maury's or Fitch's "Dental Surgery," and there seeing this work dated 1826, may have jumped to the conclusion that this account had been carried over from the first edition. This I have shown conclusively to be an error. It first appeared in the fifth edition, published in 1843. No dental bibliography that I have seen notes the various editions of this work; all note only the first or the second editions of 1826. We have evidence that Taveau called attention to the use of amalgam for filling cavities before the date of his book, and that it had been a matter of discussion among Parisian dentists, since it is mentioned in Lefoulon's work, published several years before, and also that objections which had been made to it are referred to by Taveau. Lefoulon states that it takes several days to harden, and suggests, as part of the instructions given by Taveau, that it takes about two hours' trituration to thoroughly combine the mercury and silver, and, for convenience, advises that a quantity may be mixed at a time, and kept in a glass-stoppered bottle ready for use. Taveau believed that the

---

[1] Nouveau Traite Théorique et Pratique de l'Art du Dentiste. J. Lefoulon, Paris, 1841, page 264. Bond's Translation of the same, Baltimore, 1844, page 147.

hardening of the amalgam was due to the evaporation of the mercury by the heat of the mouth. This erroneous idea continued to be entertained for many years by those who opposed its use in this country. Taft[1] says, "Amalgam fillings, in a short time after their insertion, undergo a hardening process, caused mainly by the evaporation of the mercury. The consequence is, either that the mass becomes porous or that it contracts."

The earliest published formula for preparing amalgam for dental use I have so far found is in a little work entitled "A Popular Treatise on the Structure, Diseases, and Treatment of the Human Teeth," by J. L. Murphy, London, 1837, published, it will be noticed, at about the time Taveau says he discovered the silver paste. Murphy says, discussing materials for filling cavities of decay, on page 104, "In cases where a large cavity is excavated, and the gold will not suit as a succedaneum, other material must be had; something in the shape of a paste is required. I have in cases of this kind, for many years, used an amalgam of silver, prepared in the following manner; and though there are objections against it, still, until something better is laid before the public, it will be found of a highly useful nature. The ingredients necessary for the cement are silver filings, thinnest silver-leaf (to be had in small books), and quicksilver. A drop of quicksilver is poured out from the bottle into a small mortar, or, if more convenient, on a cloth; on this is placed a leaf of silver from the book, which, being worked into the quicksilver, becomes speedily amalgamated with it; another leaf is then added, and so on until the amalgam is a thick paste; if there be too little quicksilver in this amalgam, the want will be made perceptible by its non-adhesion, and, on being worked between the fingers, by its crumbling. If, on the contrary, it possesses too much mercury, it will be too soft, and the mercury may be easily squeezed out.

"When the amalgam is brought to a proper consistency, having just sufficient mercury to enable it to be used as a paste, a small quantity of silver filings should be mixed with it: these absorb a portion of the quicksilver, and hence there is insufficient mercury left to keep the silver in a soft state; thus, after the filings are introduced, the amalgam gradually hardens. Here, then, we are in possession of a cement which may be used in a soft, cold state; yet, on being placed in the tooth, speedily hardens in the cavity."

---

[1] A Practical Treatise on Operative Dentistry, Philadelphia, 1859, page 89.

This brief notice of amalgam secured for this writer from the editor of the *American Journal of Dental Science* a rather sharp criticism,[1] for in those days a kind word for amalgam was in this country an unpardonable sin.

When, by whom, and where amalgam was first introduced into dental practice I have been unable to ascertain. Dr. J. F. Flagg, writing under date of Boston, December 5, 1843,[2] says that, under the names of royal succedaneum, enamel cement, bone paste, diamond cement, mineral paste, and lithodeon, compounds of mercury and other metals have been in use the last thirty or forty years. While we cannot regard this as an authoritative statement, it, taken with many others of like import, establishes the fact beyond dispute that it was well known long before its reputed discovery by Taveau. It is quite likely that it made its advent shortly after Regnard, in 1818, published his memoir, advising the addition of mercury to fusible metal as a means of reducing the heat necessary in packing it into properly excavated cavities of carious teeth. We have evidence that early in the forties, in England, amalgam was in use, made from alloys containing, in combination with silver, tin, copper, zinc, gold, platinum, etc. Amalgam was more kindly received in England than with us, and now and again English writers have remarked that they could not understand the unreasoning opposition to it in the United States; the "amalgam war" was a mystery that they were unable to fathom. The first real improvement known in this country—the well-known formula of four parts silver and five parts tin—drifted over from England during a correspondence preceding the first advent of crystal gold in the early fifties or before. In this way it came to the knowledge of Dr. William M. Hunter, of Cincinnati, who gave it a practical test, and, being pleased with the results, during a meeting of the American Society of Dental Surgeons, convened at Cincinnati, May 8, 1855, exhibited a number of fillings made with it in the mouths of his patients to some of the members, among others, Dr. Elisha Townsend. On his return East, Dr. Townsend, in reporting to his professional brethren upon matters brought out at this meeting, mentioned this private exhibit of amalgam fillings at Dr. Hunter's office, and repeated the formula and instructions for preparing the alloy and amalgam as given by him, always, however, scrupulously crediting the information to Dr. Hunter.

---

[1] American Journal of Dental Science, vol. ii., September, 1841, page 155.

[2] Ibid., vol. iv., March, 1844, page 210.

He was surprised at the interest it excited, and not at all pleased; for Dr. Townsend was one of those who believed that it were better that a tooth should be *lost* than be *saved* and made comfortable by amalgam, and in a communication to the profession a few months before his death took back all that he had ever said in its favor.[1] The statements now and again made, that the profession is indebted to him for an improved formula, is a pure fiction, as much so as is the statement that amalgam was introduced to the profession by O. Taveau in 1826. Both have been thoughtlessly made. Their constant repetition is not so much due, perhaps, to carelessness and indifference of dental writers as to the neglect of the profession to provide for them the means by which errors such as these may be corrected.

[1] See Dental News-Letter, vol. ix., October, 1855, page 35, and vol. xi., April, 1858, page 169.

